# Global Preparedness Against COVID-19: We Must Leverage the Power of Digital Health

**DOI:** 10.2196/18980

**Published:** 2020-04-16

**Authors:** Sultan Mahmood, Khaled Hasan, Michelle Colder Carras, Alain Labrique

**Affiliations:** 1 Digital Healthcare Solutions Dhaka Bangladesh; 2 International Health Johns Hopkins Bloomberg School of Public Health Baltimore, MD United States; 3 Epidemiology Johns Hopkins Bloomberg School of Public Health Baltimore, MD United States; 4 Health Policy and Management Johns Hopkins Bloomberg School of Public Health Baltimore, MD United States

**Keywords:** informatics, global health, developing countries, internet, infection, control, COVID-19, pandemic

## Abstract

The coronavirus disease (COVID-19) pandemic has revealed many areas of public health preparedness that are lacking, especially in lower- and middle-income countries. Digital interventions provide many opportunities for strengthening health systems and could be vital resources in the current public health emergency. We provide several use cases for infection control, home-based diagnosis and screening, empowerment through information, public health surveillance and epidemiology, and leveraging crowd-sourced data. A thoughtful, concerted effort—leveraging existing experience and robust enterprise-grade technologies—can have a substantive impact on the immediate and distal consequences of COVID-19.

## Introduction

As of April 8, 2020, the total number of confirmed coronavirus disease (COVID-19) cases rose to 1,279,722 with 72,614 deaths [1]. The outbreak that started at Wuhan city in China has now spread worldwide, and on March 11, 2020, the World Health Organization (WHO) declared COVID-19 a pandemic [2]. It threatens human lives and has disrupted global trade, travel, and employment, which risks triggering a global economic recession. China, India, and other countries have taken necessary strict measures to control the spread of the virus, which, although effective, can also lead to anxiety and the potential loss of livelihoods [3]. The situation escalated so dramatically that the Italian government was forced to change its lockdown of only the northern region to encompass the entire country [4]. For now, the main objectives of mitigation remain focused on reducing the velocity of the epidemic and minimizing the daily burden of morbidity and mortality, thus, reducing the risk of exhausting health care systems. This, in turn, helps to protect local and regional economies, keeping the epidemic and its impact manageable until a suitable antiviral drug is identified or a vaccine can be developed [5].

We have seen many guidelines for health care workers (HCWs) around preparedness for COVID-19 that focus on safety and minimizing the spread of infection. Most countries have yet to release a formal guideline or recommendation (either from the government or health agencies), which emphasizes the powerful role telemedicine and other digital health tools can play to contain and manage this new pandemic. Small but significant measures have made a difference, such as the US Office of Civil Rights and Department of Health and Human Services decision to suspend certain electronic communications privacy regulations to allow providers to support patients via commercial telehealth platforms, irrespective of those platforms compliance with health information security laws [6]. The applications of digital technology for the treatment, diagnosis, support of self-management, and surveillance during public health emergencies are well known. Many countries have existing systems in place to address a variety of health care functions without face-to-face contact [7]; the importance of taking advantage of these cannot be understated. We call for governments, health agencies, and health care providers to immediately and coherently leverage the power of digital health tools to strengthen their health care system capacity to respond to the COVID-19 pandemic. This paper presents several use cases to illustrate possible applications but is not an exhaustive or prioritized list. The use, feasibility, and importance of these applications and others will vary by country needs, existing infrastructure, and other factors.

## Stronger Infection Control Through Remote Monitoring and Training

Contagious diseases like COVID-19 pose a serious threat to HCWs and all levels of support staff who come into contact with patients. The Ebola outbreak of 2014-2016 resulted in a humanitarian crisis with more than 28,600 cases and 11,325 deaths [8]. During the height of the outbreak in August 2014, WHO reported that 240 health workers had become infected in West Africa; half of those workers lost their lives to Ebola [9]. The loss of HCWs exacerbates the situation and puts immense pressure on already fragile health systems of lower- and middle-income countries (LMIC) often struggling with limited clinical human resources to begin with.

### Teleconsultations With Early Stage or Mild COVID-19

As there are currently no curative treatments (antiviral drugs) or preventive interventions (vaccines), the recommended treatment of uncomplicated COVID-19 cases is mostly supportive with strict infection prevention and control (IPC) measures [10]. WHO has recommended that suspected COVID-19 cases with mild symptoms and no underlying problems can usually be treated at home with careful clinical monitoring [11]. However, trying to take appropriate home-based measures without clear supervision and guidance may create stress and even panic among symptomatic people who may wonder when they are “truly sick” and need to seek professional care. The use of teleconsultations is being widely recommended in most high-income contexts to protect health facilities from being overwhelmed by cases with mild to moderate illness that can be managed at home, as well as they might be in a health care facility environment. In the United States and Europe, most large health care systems have ramped up the use of existing teleconsultation offerings to their members, and a number of private sector companies offer single-use telemedicine services to uninsured or out-of-group clients.

In other contexts, creative use of commercial video conferencing platforms usually reserved for social interactions are being repurposed for clinician-patient interactions. To prevent the spread of COVID-19 to high-risk patients with other comorbidities requiring clinical follow-up, routine health care interactions are being shifted to teleconsultations in many countries around the world. In India, high-throughput tertiary referral centers such as the All India Institute of Medical Sciences are launching telemedicine services to replace in-person check-ins during the pandemic [12], and the national government has released on March 26 a set of National Telemedicine Practice Guidelines [13]. Current levels of mobile phone penetration and the level of internet connectivity allow for telemedicine solutions to be launched in most urban areas of the world. The operational cost of running a telemedicine center can be low compared to running primary care facilities of specialized hospitals with similar catchment areas, reducing the economic burden on strained health care facilities and systems [14].

### Remote Monitoring of Infection Prevention and Control

Keeping up with rapidly changing recommendations for IPC during a pandemic is challenging. As the levels of presymptomatic transmission of severe acute respiratory syndrome coronavirus 2 (SARS-CoV-2) becomes clear, we see how medical staff may be vulnerable to infection or even be at an increased risk for spreading infection in the community [15]. Doctors or other HCWs may inadvertently keep attending patients even after developing symptoms, leading to possible iatrogenic infection and quarantine for many [16]. Digital tools can play a crucial role in IPC by facilitating the monitoring and quality control of IPC practices. So far during the current outbreak, the Chinese government has reported 1716 cases among health workers and 6 deaths [17]. The number of deaths may seem low, but some studies suggest that infected HCWs may have a more severe illness [18]. To reduce the risk of hospital-based transmission, Guangdong Second Provincial General Hospital in the Guangdong province of China has implemented a proactive infection control tool that resembles a security monitoring station. At this station, highly trained infection control observers monitor IPC procedures, ensure adequate IPC supplies, and provide real time aid by radio when needed. Future plans for the system include the addition of artificial intelligence algorithms to speed the ability to identify high-risk situations and mitigate them even more quickly [19].

### Centralized Training and Capacity Building, Delivered Digitally

Preparedness activities to date have focused on training and capacity building of the health care staff on infection control, isolating infected people, and tracing contacts of suspected cases. All these tasks can be remotely managed from an appropriately staffed national-level call center. Centralizing training through the internet or mobile phones will make it easier to train staff in rural and remote areas where provider and training capacity is often variable. In this scenario, online courses such as those provided by WHO and other public domain courses for HCWs on COVID-19 can meet crucial training needs [20].

Some barriers must be overcome to ensure the benefits of digital health for remote monitoring, treatment, and training are available worldwide. In the most remote areas of the world, internet connectivity is limited and not affordable for many. Online formats are also not able to address the differences in learning style or ability that might result in deviation from standard care. In some cases, voice calling may be the most appropriate way to provide telemedicine services, such as places where internet penetration is low or with subpopulations like older adults who may not be as familiar with advanced technologies yet or are at greatest risk for critical illness. Even so, leveraging digital health for remote observation and care, where possible, may reduce costs and decrease the chances of infection spreading from patients to HCWs and back to other patients. It could also greatly help to relieve the emotional turmoil and stress of a person with a suspected case and their family members. The standard clinical recommendation in most health crises to “seek medical attention” should be, in this pandemic, “stay home if you are sick and call the coronavirus hotline to find out what to do next”. In fact, the US Centers for Disease Control and Prevention (CDC) website makes it clear that, during this pandemic, both patients with suspected COVID-19 and those with routine illnesses should first “call before you get medical care,” to prevent unnecessary exposures to others, especially care providers [21].

## Home-Based Diagnosis and Screening

Over the weekend of March 14-15, travelers returning to their home countries packed into tight queues at airports for coronavirus screening for hours. The reality of any crowded area such as testing centers is now the same; any infectious patient can transmit SARS-CoV-2 to those nearby. If a person was not infected before they went for testing, they may become infected simply by seeking testing. Testing for SARS-CoV-2 requires a minimum biosafety level (BSL)-2 to BSL-3 certified laboratory to handle infectious samples according to the newly published interim laboratory biosafety guideline from WHO [22]. Many LMIC lack these kinds of facilities. In countries where they do exist, they are usually located in major cities, which suggests that most people who may be infected cannot be tested or must travel far from home, possibly spreading infection on the way.

Home-based diagnostics can alleviate the need for suspected COVID-19 cases to travel, allowing people to continue the recommended self-quarantine. Individuals or HCWs can request a central emergency operations center hotline to deploy highly trained personnel to collect required samples or assess and transport patients to the hospital if necessary. Such an approach has been used in Milan where a specialized COVID Response Team worked with Emergency Medical Services to dispatch ambulances or test and monitor patients at home based on a procedural algorithm [23]. This centralized deployment makes sense from a resource-sparing perspective; it is obviously much easier to train a handful of sample collection personnel on infection control than trying to manage hundreds or thousands of noninfected and infected people interacting on their way to hospitals or diagnostic centers. Centralized triaging and deployment of personnel and equipment has been implemented in several countries, it is less common in the LMIC where it would preserve vital resources and act as an important form of risk mitigation.

## Empowering Through Information

Critical health advice to populations at risk changes rapidly during public health emergencies. Some actions are relatively simple, such as the WHO recommendation that all persons returning from COVID-19-affected countries stay home and self-isolate for 14 days [24]. However, despite clear guidance, ensuring that the message reaches not only public health personnel but also community leaders and members is a substantial challenge.

### Centralized Helplines for COVID-19 Information

Formal media channels such as TV, newspapers, or international and national website-based guidance provide a “firehose” of information. Helpful and reliable information may be difficult to pick out from the high-velocity stream, and people can often become confused with misinformation picked up from hyper-sensationalized fringe news outlets and myths floating around social media. Suspected cases and their caregivers may be frustrated by apparently contradicting messages or when encountering situations that are not clearly addressed by the information they have access to. Being able to communicate with a doctor or health professional trained in COVID-19 care can be reassuring and maximize the likelihood of appropriate and timely care. With a simple call to a helpline, both patients and their caregivers can be empowered with knowledge of how to minimize risk of spread, basic home care, and when to notify the health authority if the condition of a patient with COVID-19 deteriorates. It is likely more efficient to train call center–based doctors on management of suspected cases than to conduct mass communication campaigns necessary to ensure the general population has sufficient knowledge to protect themselves and their communities. Mass communication campaigns may also be ineffective in countries with low literacy rates. Another advantage of centralizing information provision is quality control; a digital knowledge base can be easily managed and updated on a regular basis as the outbreak evolves and new information and guidance emerges. With COVID-19, initial messaging around the disease’s mild manifestation in those younger than 65 years may have contributed to the widespread misinterpretation that younger subgroups of populations are immune from potentially dangerous complications; when in reality, they are only at a lower risk than older adults.

### Psychological Intervention for the Quarantined

To contain the outbreak and limit its impact, WHO and CDC experts recommend or even enforce the quarantine of infected people (ie, those who have laboratory confirmation regarding the presence of SARS-CoV-2) while suspected cases are asked to self-isolate. Today, several cities around the world are locked down, their residents prohibited from venturing outside except to visit the doctor or get food. Many governments have recommended or required suspension of mass gatherings including offices, factories, museums, schools, universities, and libraries [25]. Some cities enforce barriers to entry—no one is allowed to get into those cities unless absolutely necessary. This level of containment on a global scale is unprecedented.

These necessary but extreme measures are stressful for most and, for some, lead to panic and a loss of equanimity. News reports and social media show empty shelves in supermarkets where people depleted basic supplies like toilet paper, paper towels, cleaners, and nonperishables. These stark photos show the tenuous emotional state of those who are being asked to adopt a set of behaviors never before seen in this generation. Not surprisingly, a recently published review on the psychological impact of quarantine revealed that people in quarantine show anger, confusion, frustration, fear, and symptoms resembling post-traumatic stress disorder [26].

Asking citizens to make such sacrifices without appropriate support is unsupportable in a civilized world. We can use digital communication to deliver mental health support, provide counseling, and link individuals through online social networks. This also protects counselors and psychologists who will be able to provide support without exposing themselves to the pathogen. Digital platforms for telepsychiatry and online support are not unusual nowadays in some areas of the world, but much more could be done to leverage their unique ability to meet these needs. Many countries face severe challenges to addressing the needs for psychological support during times of crisis [27]. The addition of telemedicine means that the counsellor need not be in the same area or even in the same country. These efforts can be managed regionally or even with the minimum requirement that both the counsellor and counselee speak the same language. Some nonprofit organizations already provide peer mental health crisis support through digital platforms [28]; these models could be built on to address the needs of various populations worldwide.

Another crucial area that could be addressed through digital means is risk communication around public health measures. WHO guidelines for risk communication in public health emergencies point to the importance of unified messaging that is adaptable to local contexts [29]. People need an understandable rationale, especially in situations where longer quarantine is necessary. Various approaches can provide information and collect community feedback quite easily through digital methods without risking any healthy lives. However, one important caveat holds true for all risk communication during emergencies, as well as for any intervention in general: the acceptance and effectiveness of such measures must be rigorously evaluated, monitored, and updated as needed.

## Public Health Surveillance and Epidemiology

Digital tools can be invaluable to reduce exposure risk for public health personnel. Using a variety of remote methods, critical tasks can be performed from safe environments while gathering and analyzing the high-quality data necessary to mitigate the effects of the pandemic.

### Contact Tracing

Contact tracing is a standard procedure implemented during an outbreak to determine the extent of the outbreak by identifying and maintaining contact with persons who were exposed to a confirmed case (and thus have high probability of becoming cases themselves) [30]. Traditionally, outbreak investigators would go door-to-door to unearth detailed information of the contacts, which requires enormous amounts of time and human resources. If the disease is contagious enough, the speed of contact tracing can be outpaced by the number of cases and disease spread. Hellewell and colleagues [31] argued that the probability of controlling an outbreak through isolation of cases and contacts drops if initial numbers of cases are high, if there is higher transmission during incubation period, and if the transmissibility basic reproduction number stands between 2.5-3.5. Digital technology may solve that problem by providing a more agile and less resource-consuming approach. After confirming a case, their contacts can be traced over the phone while recording information into an electronic medical record (EMR) or contact management database. All contacts can then be followed up over time through the telephone without requiring face-to-face contact and further infection risk. Automated text message or interactive voice response systems can maintain continued contact over the relevant risk period of 7-14 days to detect early symptoms and refer sick persons to information about self-care or care-seeking, as appropriate.

In most parts of the world the health care system is pluralistic (made up of public, private, and nongovernment organization providers), and resource constraints make it difficult to regulate care. In these settings, services are often offered under the radar, and patients are free to choose and change hospitals at will, resulting in almost no ability to track who is going where. Even in the absence of an interoperable digital health information system that could facilitate disease surveillance, a centralized EMR system, accessible through a web interface or smartphone app, can facilitate the tracking of COVID-19 cases during this public health emergency.

### Leveraging Crowd-Sourced Data

So many factors can interfere with the careful public health reporting needed in epidemics: hospital channels may require excessive paperwork or administrative procedures, training may be lacking, or resources for reporting just do not exist. Right now, we need innovative solutions to ensure health care facilities can stay prepared. In a recently published article toward early analysis of the COVID-19 outbreak, Sun et al [32] showed how a health care–oriented social network can be a source of data collection and information sharing. In such scenarios, health care–oriented social network sites can provide real time data and serve as an early alarm to an imminent outbreak, which can be contained with minimal resources if timely responses can be ensured. In this recent outbreak, Chinese doctors who sounded the alarm in early January 2020 about an unusual pneumonia were later silenced with the accusation of spreading misinformation [33]. Had that alarm been taken seriously, it may have been possible to avert to some extent a global pandemic and its potential long-term health, social, and economic consequences.

Another promising project focuses on crowd sourcing artificial intelligence tasks for a database of COVID-19 research outputs [34]. The COVID-19 Open Research Dataset, a joint project of government, academic, and private institutions, compiles thousands of research articles on SARS-CoV-2 and related coronaviruses. Machine learning researchers are asked to complete and submit tasks that analyze data from the database, with research questions such as “What is known about transmission, incubation, and environmental stability?” Such challenges build on the success of previous efforts that showed that incorporation of corrected Google Flu Trends while forecasting influenza-like illness improves accuracy when compared to reporting done only through formal reporting channels [35]. As the WHO Scientific and Technical Advisory Group for Infectious Hazards has recommended to monitor public health strategies with intensified active surveillance, innovative data sources and crowdsourced tasks could add significant value [36].

However, the challenge remains to ensure the reliability and validity of data sourced through social media. Social media posts are voluntary submissions on the part of individuals and groups, and lack any sort of gatekeeping. Although it is reassuring to see that several major platforms recently decided to commit to working together to stop the spread of coronavirus misinformation, the global reach of these platforms means that this will be a difficult task [37]. Keeping this in mind, it is high time that relevant stakeholders and regulatory bodies determine how to tackle this problem in the era of the fake news and misinformation epidemic (infodemic) [37].

## Conclusion

In the midst of such a global crisis all possible opportunities need to be adequately explored and leveraged. Some countries have started taking several initiatives but often in a siloed manner. In Bangladesh, both the government and a few private organizations have started rolling out toll-free (or with minimal charge) hotline numbers for providing people with authenticated information and guidance on what to do if someone suspects they are infected by SARS-CoV-2 or actually shows symptoms of COVID-19. We also have seen the viral spread of videos with animated viruses or catchy dance steps promoting hand washing, aimed at a tech-savvy generation of connected millennials. Such measures are sporadic with likely minimal impact on the pace and consequences of this pandemic.

A thoughtful, concerted effort leveraging existing experience and robust enterprise-grade technologies can have a substantive impact on the immediate and distal consequences of COVID-19 as well as other future health care needs. Many countries have systems in place that could be leveraged in the current emergency. Building on existing infrastructure and systems will help speed digital interventions into practice and reduce costs. As we are seeing therapeutics and vaccines against this novel coronavirus being fast-tracked, so too must we identify and accelerate the use and adoption of digital strategies such as those described in this paper. Normative agencies like WHO can help rapidly convene the expertise needed to develop the content (eg, decision logic and workflows) and data models (eg, recommended variable types and names) that would help developers expedite locally appropriate solutions that are built on validated content but also interoperable—allowing deidentified data to be rapidly pooled as part of global efforts to understand emergent pandemic threats. Unlike the 1918 influenza pandemic, which claimed an estimated 50 million lives, we are confronting COVID-19 within the context of a digital, connected planet. Digital health solutions have been reviewed and vetted by global health agencies like WHO [7] and are available to be deployed in short order through public-private partnerships. This is only possible if we move quickly, like we have for other conventional mitigation strategies, to approve their use to prepare, detect, contain, and better understand this daunting pathogen.

## Abbreviations

BSL: biosafety level
CDC: Centers for Disease Control and Prevention
COVID-19: coronavirus disease
EMR: electronic medical record
HCW: health care worker
IPC: infection prevention and control
LMIC: lower- and middle-income countries
SARS-CoV-2: severe acute respiratory syndrome coronavirus 2
WHO: World Health Organization
